# On the Role of Paraoxonase-1 and Chemokine Ligand 2 (C-C motif) in Metabolic Alterations Linked to Inflammation and Disease. A 2021 Update

**DOI:** 10.3390/biom11070971

**Published:** 2021-07-01

**Authors:** Jordi Camps, Helena Castañé, Elisabet Rodríguez-Tomàs, Gerard Baiges-Gaya, Anna Hernández-Aguilera, Meritxell Arenas, Simona Iftimie, Jorge Joven

**Affiliations:** 1 Unitat de Recerca Biomèdica, Hospital Universitari de Sant Joan, Institut d’Investigació Sanitària Pere Virgili, Universitat Rovira i Virgili, 43201 Reus, Spain; helena.castane@urv.cat (H.C.); elisabet.rodriguez@urv.cat (E.R.-T.); gerard.baiges@iispv.cat (G.B.-G.); anna.hernadez@grupsagessa.com (A.H.-A.); marenas@grupsagessa.com (M.A.); 2 Department of Radiation Oncology, Hospital Universitari de Sant Joan, Institut d’Investigació Sanitària Pere Virgili, Universitat Rovira i Virgili, 43201 Reus, Spain; 3 Department of Internal Medicine, Hospital Universitari de Sant Joan, Institut d’Investigació Sanitària Pere Virgili, Universitat Rovira i Virgili, 43201 Reus, Spain; smiftimie@grupsagessa.com

**Keywords:** cancer, cardiovascular disease, chemokine (C-C motif) ligand 2, fatty liver, infection, inflammation, metabolism, obesity, paraoxonase-1

## Abstract

Infectious and many non-infectious diseases share common molecular mechanisms. Among them, oxidative stress and the subsequent inflammatory reaction are of particular note. Metabolic disorders induced by external agents, be they bacterial or viral pathogens, excessive calorie intake, poor-quality nutrients, or environmental factors produce an imbalance between the production of free radicals and endogenous antioxidant systems; the consequence being the oxidation of lipids, proteins, and nucleic acids. Oxidation and inflammation are closely related, and whether oxidative stress and inflammation represent the causes or consequences of cellular pathology, both produce metabolic alterations that influence the pathogenesis of the disease. In this review, we highlight two key molecules in the regulation of these processes: Paraoxonase-1 (PON1) and chemokine (C-C motif) ligand 2 (CCL2). PON1 is an enzyme bound to high-density lipoproteins. It breaks down lipid peroxides in lipoproteins and cells, participates in the protection conferred by HDL against different infectious agents, and is considered part of the innate immune system. With PON1 deficiency, CCL2 production increases, inducing migration and infiltration of immune cells in target tissues and disturbing normal metabolic function. This disruption involves pathways controlling cellular homeostasis as well as metabolically-driven chronic inflammatory states. Hence, an understanding of these relationships would help improve treatments and, as well, identify new therapeutic targets.

## 1. Oxidation, Inflammation and Disease

Tissues produce reactive oxygen species (ROS) as a metabolic by-product in response to environmental factors such as an imbalanced diet or an infectious process. ROS react with lipids, proteins, and nucleic acids and result in alterations in cell structure and function [1]. The organism has enzymatic and non-enzymatic antioxidants to block the harmful effects of ROS. However, these protective systems can be overwhelmed in disease states. An inflammatory reaction is generated when oxidative equilibrium is disrupted. For example, in infectious diseases, ROS production by host macrophages is an important part of the defense mechanism against infecting bacteria or viruses, and the imbalances produced can trigger an inflammatory reaction [2]. In turn, inflammation can lead to a further increase in oxidative stress and thus enter a vicious cycle that can aggravate the disease [3,4]. Independently of whether oxidative stress and inflammation represent the causes or the consequences of cellular alterations, an overwhelming amount of evidence indicates that both processes contribute to the pathogenesis of many diseases. The role played by macrophages and their polarization need to be considered in detail in the setting of chronic inflammatory diseases [5]. These diseases are associated with an increase in M1, or “classically activated” macrophages, and a decrease in M2, or “alternatively activated” macrophages [6,7,8]. Chemokines are involved in macrophage polarization, while directing the traffic of immune cells to sites of inflammation and activating the production and secretion of inflammatory cytokines [9]. Monocytes migrate to the site of inflammation and differentiate into macrophages when the chemokine (C-C motif) receptor (CCR2) interacts with the chemokine (C-C motif) ligand 2 (CCL2): a key process in the development of inflammatory diseases [10]. Recent studies indicate that other chemokines and chemokine receptors also play important roles. For example, the CCL5/CCR5 complex has been related to cancer and infection [11,12,13,14].

## 2. Paraoxonase-1 Is an Antioxidant Enzyme That Participates in the Innate Immune System

Paraoxonase-1 (PON1) belongs to an enzyme family composed of three members (PON1, PON2, and PON3), which are the protein products of a gene that evolved by duplication of a common precursor. They have high structural homology with each other [15,16], and the three genes are located in adjacent positions of chromosome 7 (7q21.3) [15,16]. PON1 is a lactonase and an esterase that catalyzes the hydrolysis of thiolactones, organophosphate esters, unsaturated aliphatic esters, aromatic carboxylic esters, and carbamates [17,18]. PON2 and PON3 do not degrade esters but have lactonase activity [19]. PON1 and PON3 degrade lipid peroxides in low-density lipoproteins (LDL) and high-density lipoproteins (HDL) [19]. In humans, PON1 and PON3 are mainly synthesized by the liver, and the enzymes are found in blood bound to HDL [20,21,22]. The enzymatic action of PON1 is exerted in the circulation within HDL particles, but they can also be transported from these particles to the cell membranes [23], especially of epithelial and endothelial cells [24,25,26]. Conversely, *PON2* gene expression is exclusively intracellular [27].

In addition to degrading oxidized lipids, PON1 inhibits CCL2 synthesis [28,29]. In vitro treatment of oxidized LDL with purified PON1 reduces the degree of lipid oxidation and the ability of this lipoprotein to induce interactions between monocyte and endothelial cells [30]. Further experimental studies have shown that HDL particles obtained from *PON1* deficient mice lacked the ability to protect LDL from peroxidation [31], and that *PON1* and *apolipoprotein-E* double deficient mice had higher levels of lipid peroxidation products in vivo than the animals that were deficient in *apolipoprotein-E* alone [32].

PON1 can also protect the organism from bacterial biofilm formation through its lactonase activity [33]. A biofilm is an aggregation of bacteria, embedded within a matrix of polysaccharides, proteins, and DNA. Being embedded within biofilms provides bacteria with protection and, by developing antibiotic resistance, can make successful treatment difficult [34,35,36]. Quorum sensing is essential for the formation of biofilms. This phenomenon is defined as the coordination of bacterial behavior through the accumulation of signaling molecules, i.e., when the concentrations of signaling molecules reach a critical threshold, the result is modulation of certain target genes that trigger the formation of biofilms [37]. In Gram-negative bacteria, *N*-acyl-homoserine lactones (AHL) have been identified as the major signaling molecules in this communication system [38,39,40]. Considerable evidence suggests that PON1 plays an important role against biofilm formation. Lung epithelial cells and resident macrophages are important defense mechanisms against airborne microorganisms. PON1 protein is strongly expressed in lung epithelial cells and, as described above, it has lactonase activity [25,26]. Thus, it seems logical to infer that PON1 is able to hydrolyze AHL and interrupt quorum sensing signals. This hypothesis was demonstrated by investigators who found that a lactonase present in lung epithelial cells inactivates 3-oxo-C12-AHL [41,42,43,44]. These researchers also reported that wild-type mouse serum, rich in PON1, degraded 3-oxo-C12-AHL and decreased *P. aeruginosa* biofilm formation, and that this capacity was lost when serum from *PON1* deficient mice was employed instead. In addition, adding purified PON1 to serum from *PON1* deficient mice restored the ability to degrade 3-oxo-C12-AHL and inhibited biofilm growth [44]. These data demonstrate that PON1 efficiently degrades 3-oxo-C12-AHL and reduces the growth of bacterial biofilms. Currently, it is widely accepted that PON1 is an enzyme with multiple hydrolytic capacities causing lipoperoxide degradation and counteracting oxidative stress in the circulation and within cells. PON1 also inhibits the synthesis of CCL2 and the subsequent inflammatory reaction, and protects against the formation of bacterial biofilms. Overall, these results indicate that PON1 can be considered part of the organism’s innate immunity system [45].

## 3. The Protective Role of Paraoxonases on CCL2 Expression, Mitochondrial Function, and Metabolism

Several lines of research suggest that the PON family of enzymes play a prominent role in the protection of cells against mitochondrial dysfunction and metabolic alterations. Most studies in this field have been conducted on PON2 because the general consensus is that this is the major intracellular enzyme. For example, PON2 has been reported to reduce the unfolded protein response (UPR) accompanying oxidative stress and UPR-derived caspase activation in human vascular cells [27,46,47]. In addition, the expression of genes related to endoplasmic reticulum stress was increased in macrophages from *apolipoprotein E* and *PON2* double deficient mice, compared to those that were only *apolipoprotein E* -deficient [48]. These authors observed that treatment of macrophages from *apolipoprotein E* and *PON2* double deficient mice with an inducer of endoplasmic reticulum stress resulted in mitochondrial dysfunction, increased oxidative stress, and increased cell apoptosis. Further studies in several experimental models have added more evidence that PON2 protects mitochondrial function and prevents apoptosis [49,50,51].

**Figure 1:**
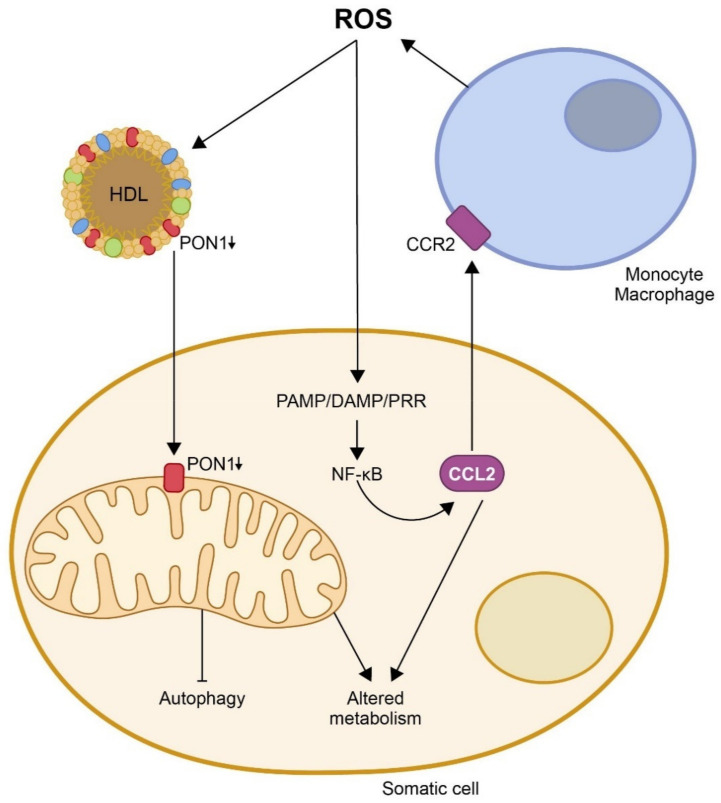
**Oxidation, inflammation, and disturbances in energy metabolism are closely related**. To date, the evidence reported suggests that excessive production of reactive oxygen species (ROS) would inhibit paraoxonase-1 (PON1) activity in high-density lipoprotein (HDL) particles and in the mitochondrial membranes of somatic cells. At the same time, it would stimulate the synthesis of chemokine (C-C motif) ligand 2 (CCL2) through several pathways, notably that of pathogen-associated molecular patterns/damage-associated molecular patterns/pattern-recognition receptors (PAMP/DAMP/PRR). The decrease in PON1 activity and the increase in CCl2 would cause alterations in mitochondrial metabolism and an inhibition of autophagy. At the same time, CCL2 would interact with its receptor (CRR2) and present on monocytes, promoting their migration to sites of injury, their differentiation to macrophages, and their synthesis of new ROS, producing a vicious circle that would trigger and aggravate the disease.

From the above-mentioned data, it is inferred that PON2 does indeed play a protective role in mitochondrial function, but this is not so clear regarding PON1. Data suggest that this enzyme also protects cells from oxidation, and that this effect involves the inhibition of CCL2 synthesis. Studies from our research group have shown that *PON1* deficient mice fed an atherogenic diet had increased hepatic fat depots and a marked depression of the tricarboxylic acid (TCA) cycle. In addition, the hepatic concentrations of several markers of oxidative stress and CCL2 expression were increased [52]. Further experimental data [53] showed that dietary fat caused liver steatosis, oxidative stress, and the accumulation of pro-inflammatory macrophages in the livers of *LDL-receptor* and *PON1* double deficient mice, together with alterations in energy metabolism, in the methionine cycle, in the glutathione reduction pathway, and autophagy. Conversely, when we established a line of *LDL-receptor*, *PON1*, and *CCL2* triple deficient mice, we observed that the deletion of this chemokine normalized the metabolic disturbances and increased lysosome-associated membrane protein 2 expression, which suggests enhanced chaperone-mediated autophagy. In humans, studies have observed that individuals with obesity have impaired PON1 activity and impaired mitochondrial function [54,55]. Our group has a special interest in evaluating the hepatic alterations in patients with morbid obesity treated with bariatric surgery and, as well, in observing the metabolic effects of the treatment. To date, results have shown that one-year post-surgery, the hepatic histology of all patients was improved, especially in those who had severe steatohepatitis, bridging fibrosis, and/or cirrhosis. Additionally, we observed pre-surgery differences in plasma and liver markers of oxidative stress and inflammation (including CCL2 and PON1), which were corrected one-year post-surgery [56]. In addition, patients with steatohepatitis presented pre-surgery alterations in energy metabolism, especially in plasma concentrations of α-ketoglutarate and oxaloacetate, which reverted one-year post-surgery [57]. Overall, these results suggest an entanglement of PON1 and CCL2 in the regulation of metabolism and mitochondrial function in the liver of experimental animals, and in humans (Figure 1).

## 4. Mechanism of Action of CCL2 in the Immune Response and Inflammation and Its Relationship with Multiple Metabolic Alterations

One of the proposed mechanisms by which oxidative stress would enhance the inflammatory response is the induction and assembly of multiprotein complexes called inflammasomes. ROS activate the NOD-like receptor Pyrin domain 3 (NLRP3) in macrophages, triggering the formation of inflammasomes and an immune reaction that involves the synthesis of pro-inflammatory chemokines, from which CCL2 is, probably, the most representative [58,59,60,61]. This chemokine is upregulated following tissue injury and is expressed by both inflammatory and stromal cells. CCL2 has been reported to promote endoplasmic reticulum (ER) stress and autophagy and to regulate NF-ĸB expression by catalyzing de-ubiquitination [62]. The main pathway triggering inflammation is, probably, the activation of pattern-recognition receptors (PRRs), which recognize pathogen-associated molecular patterns (PAMPs), synthesized as a response to pathogens, or damage-associated molecular patterns (DAMPs), which are products of damaged cells [63,64,65]. The binding of PAMP/DAMP to a PRR leads to NF-ĸB activation and the production of adhesion molecules and chemokines that lead to infiltration of immune cells into the sites of tissue damage [66]. Other alternative pathways also result in similar outcomes, particularly the phosphoinositide 3-kinase-related signaling pathway, the mitogen-activated protein kinase pathway, and the Janus kinase/signal transducers and activators of transcription signaling pathway [67,68,69]. These changes induce the UPR, essentially by three ER-related transmembrane proteins, i.e., the inositol-requiring enzyme 1, the protein kinase RNA-like endoplasmic reticulum kinase, and the activating transcription factor 6 [70,71,72,73]. CCL2 and other chemokines, together with oxidative stress, trigger ER stress. In addition, the UPR may regulate inflammation through several pathways, such as the regulation of oxidative stress or the upregulation of CCR2 expression [74]; the UPR links ER stress with cell death and autophagy [75]. When cell damage is moderate, autophagy helps cells survive the injury, allowing them to heal and thus preventing cell death by removing toxic protein aggregates. However, when cell damage is high, the result is a non-apoptotic form of cell death that can be detrimental. The role of autophagy in the maintenance of mitochondrial integrity seems to be paramount [76]. Mitophagy increases cell lifespan, while repression of autophagy reduces lifespan. Several studies have linked mitochondrial dysfunction, autophagy, and age-related diseases with the activity of the inflammasomes [77,78,79,80]. Taken together, these results define a clear relationship between oxidative stress, chemokines, and mitochondrial impairment, resulting in metabolic alterations and their involvement in diseases.

Activation of the immune response and chronic inflammation has been associated with aging and age-related diseases [81,82,83]. Senescent cells secrete chemokines, which influence the trafficking of immune cells [84,85]. Epidemiological studies have suggested that CCL2 levels are increased in older individuals, independently of metabolic alterations. Moreover, in vitro studies have shown that chemokines appear to confer senescence to neighboring normal cells in an autocrine and paracrine fashion [86,87,88]. A recent study by our research group in mice with accelerated aging is a good example of such relationships [89]. We crossbred mice that overexpressed CCL2 with progeroid mice bearing a mutation in the *lamin A* gene. Wild-type animals and progeroid mice not overexpressing CCL2 were used as controls. We observed that progeroid mice lost weight (relative to the wild-type animals) and developed lordokyphosis and lipodystrophy. The lifespan was significantly reduced in both strains of progeroid mice, but this reduction was higher in those overexpressing CCL2. These mice also presented specific characteristics of metabolic dysregulation in skeletal muscle, including alterations in the glucose and TCA cycles, and in one-carbon metabolism. These data suggest that mitochondrial metabolites play major roles in pathological aging. Consequently, we investigated the mitochondrial respiratory complexes in skeletal muscle, and observed that the expressions of complexes I and V were lower in mice overexpressing CCL2. In addition, the protein concentrations of translocase of outer membrane 20 (TOM20) and mitofusin 2 (MFN2) in the muscles of the progeroid mice were decreased, indicating alterations in the correct formation of the mitochondrial network. We also observed an increase in p53, which would indicate the triggering of aging through a p53-mediated transcriptional program involving the mechanistic target of rapamycin. Indeed, we found inhibition of phosphorylation of phosphoinositide 3-kinase, indicating a mechanistic target of rapamycin inhibition. Finally, the higher microtubule-associated proteins 1A/1B light chain 3B (LC3) II/I ratio, and lower lysosome-associated membrane protein 2 (LAMP2A) and sequestosome 1 (p62) expressions suggested the involvement of chaperone-mediated autophagy as a consequence of the CCL2 overexpression in the progeroid animals.

Following on from the actions described above, in many infectious and non-infectious diseases, the adaptive immunity deteriorates, whereas the innate immunity is more responsive to stimuli; the consequence is the development of an inflammatory reaction. Several studies have linked the activation of NLRP3, which is dependent on increased generation of free radical species by mitochondria, with metabolic disturbances [90,91,92]. Hence, it is of note that awareness of the origin of free radicals and the putative mechanisms of prevention (i.e., PON1) is critical when establishing possible therapeutic interventions in order to preempt an inflammatory reaction. Within this context, the interaction of PON1 with CCL2 can play a key role.

In the following sections we summarize the current knowledge on the roles that PON1, CCL2, and metabolic alterations play in some of the more frequently-occurring diseases.

## 5. Obesity and the Associated Liver Disease

The prevalence of obesity has increased in recent decades, and the phenomenon constitutes a serious health problem worldwide [93]. Non-alcoholic fatty liver disease (NAFLD) is an important comorbidity of obesity. The most severe form of NAFLD is non-alcoholic steatohepatitis (NASH), which is often the main clinical reason for liver transplantation [94]. Excessive and unbalanced nutrient intake is a fundamental contributor to obesity, and related metabolic liver disease. Several mechanisms have been proposed to explain the metabolic alterations resulting from excessive food consumption and obesity. The capacity of adipose tissue to store and process lipids is limited and, when this limit is exceeded, adipocytes exhibit several signs of stress linked to metabolic dysfunction. Among these factors are: free radical production, mechanical stress, ER stress, hypoxia, mitochondrial dysfunction, altered chemokine and adipokine signaling, and inflammation [95,96]. Altered concentrations of circulating cytokines and chemokines are strongly associated with obesity [97,98,99,100,101]. In obese mice, all cell types within adipose tissue could, potentially, secrete cytokines. Increased expression of inflammatory mediators has been observed in the visceral fat of individuals with obesity [102,103]. Other metabolic tissues, apart from adipose tissue, contribute to the severity of the disease and, in consequence, to macrophage trafficking and infiltration mediated by chemokines [99,104,105]. Generally, the concept of abnormal nutrient intake should consider not only the total amount of calories consumed, but also their quality. Recently we reported that normal mice fed a high-fat diet develop hepatic steatosis, but if they were fed an isocaloric diet rich in fat and sucrose they developed NASH [106]. This derangement was accompanied by oxidative stress and an increase in the hepatic expression of CCL2. Additionally, we observed alterations in the TCA cycle, glycolysis, and amino acid and pentose-phosphate pathways in the liver, as well as an increase in autophagy. We concluded that, overall, the addition of sucrose to a high-fat diet promotes an oxidative and inflammatory environment, and also negates the ability to restore damaged hepatic mitochondria.

**Figure 2:**
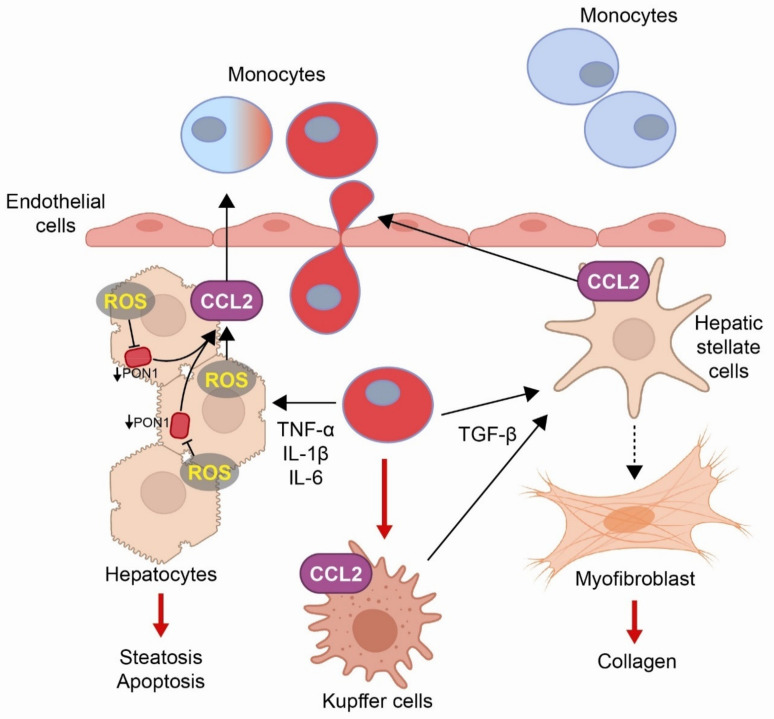
**Role of the chemokine (C-C motif) ligand 2 (CCL2) in the development of liver disease**. Reactive oxygen species (ROS) and decreased paraoxonase-1 (PON1) activity increase the synthesis of CCL2, which activates monocytes that cross the endothelial barrier by diapedesis and differentiate into macrophages. These cells synthesize pro-inflammatory cytokines, such as tumor necrosis factor-α (TNF-α), interleukin 1β (IL-1β), and interleukin 6 (IL-6), contributing to fat accumulation and apoptosis of hepatocytes. Further, monocytes and macrophages synthesize tumor growth factor-β (TGF-β) that induces differentiation of stellate cells into myofibroblasts that synthesize collagen.

Abdominal obesity, whether in adults or children, is a risk factor for metabolic syndrome and, within which, is the reduction if serum PON1 activity [107,108], while exercise increases the activity [109,110,111]. In sedentary children and adolescents with obesity, we found that decreased PON1 activities were associated with hyperinsulinemia and insulin resistance, as well as higher triglycerides and lower HDL-cholesterol concentrations. This suggested that PON1 may be involved in the metabolic alterations leading to the future development of diabetes mellitus and/or cardiovascular disease [107]. Serum PON1 activity is low in chronic liver diseases [112]. Oxidative stress and decreased PON1 activity result in an increased production of pro-inflammatory cytokines such as CCL2 and tumor necrosis factor-α (TNF-α) [112]. In patients with liver impairment, the circulating levels of these cytokines correlate with the severity of the hepatic inflammation [113,114], while the pharmacological inhibition of CCL2 results in improved liver function [115]. The role of CCL2 in the development of liver disease is schematized in Figure 2.

In humans, body mass index correlates well with adipose tissue CCL2 expression [116,117]. CCL2 has been suggested to influence the function of adipocytes and to be the link between adipose tissue inflammation, insulin resistance, and liver impairment [99,118,119]. The inflammatory reaction induced by CCL2 could contribute to deterioration of cell homeostasis and energy requirements in metabolic organs [53,118]. A recent study reported that the overexpression of CCL2 in mice was associated with increased liver and decreased muscle weights and, as such, mimicked a phenotype frequently found in obesity, liver disease, and aging [120]. The animals also displayed distinct alterations in the liver and muscles, including concentrations of metabolites from energy and one-carbon metabolism, mitochondrial fusion, and autophagy. The study concluded that mice overexpressing CCL2 had an anabolic profile in the liver, with decoupling of oxidative phosphorylation components, and alterations in mitochondrial fusion; a phenomenon related to liver disease [121,122]. Of note is that skeletal muscle had a different, catabolic, profile, with increased expression of oxidative phosphorylation components and increased levels of lactate and ketone bodies, without alterations in mitochondrial fusion markers.

Bariatric surgery is a common treatment in patients with morbid obesity, and offers a unique opportunity to investigate the metabolic derangements associated with NAFLD, especially when comparing the data obtained from a peri-surgical liver biopsy with those obtained from a percutaneous biopsy obtained post-surgery [123]. Recent results from our research group [124,125,126,127] showed that, one year post-bariatric surgery (laparoscopic sleeve gastrectomy), the prevalence of diabetes, hypertension, and NAFLD significantly decreased in patients with pre-surgery morbid obesity; the improvements in hepatic histology and function were greater in patients with NASH. We found significant pre-surgery differences in liver markers of oxidative stress and inflammation (including CCL2 and PON1) between patients with, and those without, NASH, which suggested a regulatory role of mitogen-activated protein kinases. In addition, we observed an alteration in the mitochondrial function associated with a dysregulation of glutaminolysis and increased hepatic and plasma concentrations of α-ketoglutarate. Bariatric surgery was associated with consistent improvements in these parameters. These changes influenced the adenosine monophosphate-activated protein kinase/mammalian target of the rapamycin-driven pathways that modulated hepatocyte survival by coordinating apoptosis and autophagy. Finally, we reported that α-ketoglutarate and the associated metabolites affected methylation-related epigenomic remodeling enzymes. Integrative analysis of hepatic transcriptome signatures and differentially-methylated genomic regions distinguished patients with NASH from those without.

## 6. Cardiovascular Diseases

Atherosclerosis and cardiovascular diseases (CVD) are closely associated with obesity [128]. Perivascular inflammation plays a major role in the onset and development of atherosclerosis [129,130]. Indeed, experimental studies in apolipoprotein E-deficient mice reported that perivascular inflammation precedes oxidative stress and endothelial dysfunction [129]. This experimental model is characterized by increased production of chemokines such as CCL2 [131], macrophage inflammatory protein 1-α (MIP-1α or CCL3), and CCL5 [132]. A considerable body of evidence indicates that activation of the CCL2/CCR2 axis is important in the pathogenesis of atherosclerosis [133,134]. Lipid peroxides, interleukins, angiotensin II, homocysteine, activated platelets, and shear stress, among other mediators of atherosclerosis, induce CCL2 synthesis and secretion by endothelial cells and smooth muscle cells [134]. Increased CCL2 expression has been found in macrophage-rich atherosclerotic lesions [135]. Moreover, in autopsy-derived arterial specimens from patients, CCL2 was shown to be present in the early phases of atherosclerosis; the suggestion being that this chemokine contributes to the early influx of monocytes into the vessel wall [136]. In human carotid endarterectomy specimens, a high CCL2 expression was observed in macrophage-rich areas bordering the necrotic lipid core of the atheromatous plaque; the implication being that chronic monocyte infiltration and lipid accumulation promoted by CCL2 contribute to plaque vulnerability [137]. A study by our research group [138] showed that CCL2 (observed with immunohistochemical staining), was 9-fold higher in coronary arteries obtained by autopsy from patients with coronary artery disease, compared to those of healthy individuals who died in a traffic accident. CCL2 expression was observed in only 26% of normal arteries, and was mostly restricted to smooth muscle cells with an almost negligible staining in the intima and adventitia. Conversely, CCL2 was detected in all specimens of affected arteries, and in all arterial layers, particularly those of the adventitia. Moreover, the quantitative measurement of CCL2 expression discriminated healthy artery tissue samples from that of coronary artery disease samples in a mild, moderate, or severe state with >85% sensitivity and specificity. High plasma CCL2 concentrations have been shown to be associated with increased long-term risk of stroke [139,140], while CCL2 signaling pathways have been shown to be responsible for ischemic stroke progression and atrial fibrillation [141,142].

**Figure 3:**
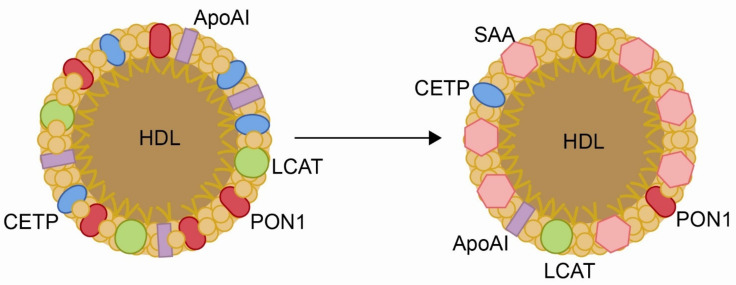
**Changes in the structure of high-density lipoproteins (HDL) produced by inflammation**. Chronic inflammatory processes cause a decrease in the content of paraoxnase-1 (PON1), apolipoprotein AI (Apo AI), lecithin:cholesterol acyltransferase (LCAT), and cholesterol ester transfer protein (CETP), and an increase in the concentration of serum amyloid A (SAA).

In the above sections, we have discussed the close relationships between increased concentrations of CCL2, oxidative stress and decreased PON1 activity. Low PON1 activities are known to increase oxidative stress as well as CCL2 synthesis and the generation of inflammatory processes. However, the opposite is also true. For example, chronic inflammation causes profound changes in the structure and in the circulating levels of HDL, among which are decreases in concentrations of PON1, apolipoprotein AI, lecithin:cholesterol acyltransferase, and cholesterol ester transfer protein, together with an increase in the concentration of serum amyloid A (Figure 3). All these changes influence the intracellular metabolism of cholesterol and increase the risk of CVD [143,144,145].

The mechanisms by which the decrease in PON1 concentrations associated with chronic inflammation can promote pro-atherogenic changes in the arterial system have received considerable research attention recently. The association of PON1 with HDL particles facilitates the binding of the particles to macrophages and, subsequently, the enzyme can hydrolyze membrane phospholipids to generate lysophosphatidylcholine, which regulates the expression of cholesterol transport proteins. Further, PON1 inhibits the formation of free radical species in macrophages by preventing the activation of NADPH oxidase and by stabilizing mitochondria. PON1 also promotes the differentiation of macrophages into an anti-inflammatory phenotype [146]. An interesting study [147] demonstrated that genetically reduced PON1 concentration induces proatherogenic changes in plasma proteomes in humans and mice. The study investigated the influence of the least efficient isoforms of *PON1* genetic polymorphisms in humans and *Pon1*−/− genotype in mice and found that both genetic modifications induce changes in the plasma proteome that affect biological networks involving proteins participating in lipoprotein metabolism, in CVD in neurological diseases, in immune response, inflammatory response, in cell-to-cell signaling, and immune-cell trafficking. In general, clinical studies have found low circulating PON1 activities in patients with CVD [148,149,150,151,152]. However, its usefulness as a risk marker has not been clearly demonstrated. A recent trial (the Dutch PREVEND study) reported an association between low serum PON1 activities in 7766 subjects with high HDL and C-reactive protein levels [153], but this association was not sufficiently robust in the general population when adjusted for other confounding factors [154].

Metabolomics has enabled the identification of metabolic alterations in patients with CVD. In addition, associated comorbidities (diabetes, obesity, metabolic syndrome) can modify systemic and myocardial metabolism and worsen cardiac function [155,156,157,158]. The earliest study conducted almost 20 years ago was promising, but the low number of cases precluded definitive conclusions [157]. More recently, a lipidomics analyses in a population-based 10-year follow-up study in Sweden showed an association between CVD and lysophosphatidylcholine, monoglycerides, and sphingomyelin, independently of traditional risk factors. Of note is that these alterations were associated with biochemical markers of oxidative stress and inflammation [159]. Another lipidomics population-based study in Italy showed that eight lipid classes (cholesterol esters, lysophosphatidylcholines, lysophosphatidylethanolamines, phosphatidylcholines, phosphatidylethanolamines, phosphatidylserines, sphingomyelins, and triacylglycerols) predict future development of myocardial infarction, stroke, and sudden death [160]. Some patient-based studies have also been conducted. Shah et al. [161] studied 2023 consecutive patients undergoing cardiac catheterization and found that five different metabolite classes were independently associated with mortality; these were branched-chain amino acids (BCAA), dicarboxylacylcarnitines, fatty acids, long-chain dicarboxylacylcarnitines, and medium-chain acylcarnitines. Of those, three lipid profiles significantly predicted fatal events, independent of other standard predictors. More recent studies found that the serum concentrations of several energy balance-related metabolites are increased in patients with dilated cardiomyopathy [162], and alterations in NO metabolism were shown to be associated with severe aortic stenosis [163].

Our research group has paid special attention to investigating the potential utility of CCl2, PON1, and associated metabolic alterations as markers in the diagnosis of peripheral arterial disease (PAD) of the lower extremities. This disease is a frequent complication of diabetes mellitus and, if not treated expeditiously, may require amputation of the affected limb. Unfortunately, the disease progresses silently and, although preventive measures are effective in early stages, PAD is often under-diagnosed when asymptomatic and, consequently, prevention is either applied too late or not at all. Therefore, the search for laboratory-measured surrogates has an evident clinical interest. Furthermore, a characteristic of PAD is that the extent of the affected area is much greater than that of vascular diseases of the upper trunk, with which the biochemical alterations measured in the circulation are potentially greater [164]. Our studies showed that patients with PAD had decreased serum PON1 activities and increased CCL2 concentrations [165,166,167], together with significant alterations in energy metabolism, including decreased circulating levels of branched-chain amino acids and increased levels of glutamate, glutamine, and several metabolites of the TCA cycle. Additionally, we found that CCL2, isocitrate, and glutamate had a high diagnostic accuracy in predicting PAD, with areas under the curve of the receiver operating characteristics (AUROC) plots > 0.95 [168]. These studies not only served to illustrate the close relationships between PON1, CCL2, and energy metabolism in inflammatory diseases, but also served to highlight new biological markers for the early diagnosis of PAD.

## 7. Cancer

Obesity is a long-term risk factor for cancer, and both disorders share deviations in common metabolic pathways [169]. Cancer is associated with oxidative stress. Experimental studies have reported that increased production of ROS by cancerous cells can cause tumor proliferation, promotion of genetic instability, and alterations in cellular sensitivity to chemotherapy [170]. Currently, serum PON1 activity appears to be decreased in many types of cancers, and this topic has been the subject of a recent meta-analysis [171]. Our research group observed decreased PON1 activities in patients with cancers of the breast, lung, head and neck, and rectum who, fully or partially, recovered post-radiotherapy [172,173,174]. Some studies investigated the relationships between serum PON1 activities and tumor stage, and reported that local progress of the disease was associated with lower enzyme activities in patients with ovarian [175] and gastroesophageal cancers [176]. However, other studies did not find any significant differences in PON1 activities in relation to the presence/absence of metastases [176,177,178] and, as well, did not find any significant associations between PON1 concentrations and tumor histology stage or location [176,179,180].

Consistent with what has been highlighted in this review is that decreased serum PON1 activity is associated with inflammation in cancer. For instance, in gastroesophageal cancers, the decrease in PON1 activity correlates directly with the levels of circulating inflammation markers, including C-reactive protein and interleukin-6 [179,181]. Additionally, we have observed low serum PON1 activities and high CCL2 concentrations in patients with bladder cancer [182]. CCL2 and other pro-inflammatory cytokines and chemokines released from tumor or stromal cells act in autocrine and paracrine modes to induce changes in tumor cells, recruit bone marrow-derived cells, favor epithelial-mesenchymal-transition, and form an inflammatory milieu that favors metastatic cell growth [183]. For instance, interleukin-1β has been shown to promote migration and proliferation of HeLa cells by targeting the NF-κB/CCL-2 pathway [184], while CCL2 was reported as promoting prostate cancer metastasis [185]. Further, the CCL2/CCR2 axis has been associated with breast cancer (BC) progression [186], and miR-196a, which activates CCL2, also promotes the migration and invasion of the lung by cancer cells [187]. In addition, high plasma CCL2 concentrations are associated with poorer response to neoadjuvant radiochemotherapy in patients with colorectal cancer [188], and with increased risk of prostate cancer [189]. All these data suggest that the CCL2/CCR2 axis could be a promising target for cancer treatment and prevention.

**Figure 4:**
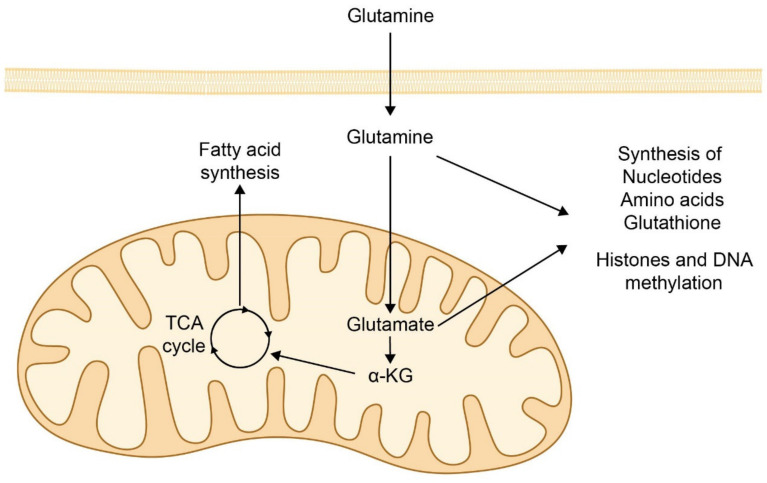
**Glutaminolysis in cancer**. Cancer cells require glutamine from the circulation to convert it into glutamate and α-ketoglutarate, (α-KG) which enter the tricarboxylic acid (TCA) cycle. These metabolites induce the synthesis of proteins, glutathione, and fatty acids, as well as epigenetic changes and metabolite exchange between the mitochondria and the cytosol.

Cancer cells also have profound changes in energy metabolism. Normal cells mainly produce energy via oxidative phosphorylation in the mitochondria. However, most cancer cells produce energy through an enhanced form of glycolysis, followed by lactic acid fermentation. Aerobic glycolysis is less efficient than oxidative phosphorylation in terms of ATP production, but aerobic glycolysis facilitates increased production of other metabolites that are required for the synthesis of lipids, proteins, and nucleic acids, which are factors in the proliferation of tumor cells [190]. This is termed the “Warburg effect” as a tribute to Otto Warburg who first described this phenomenon in 1956 [191]. Many studies have highlighted the importance of the glutaminolysis pathway in converting glutamine to glutamate and α-ketoglutarate for entry into the TCA cycle. Tumor cells convert 90% of glucose and 66% of glutamine into lactate and alanine. Glutamine and glutamate contribute to the carbon backbone in the TCA cycle, and this is relevant in conditions of carbon diversion to glycolysis [192,193]. The transfer of an amino group from glutamate to oxaloacetate via aspartate aminotransferase results in α-ketoglutarate and aspartate, whereas nitrogen transfer from glutamate to pyruvate via alanine aminotransferase results in α-ketoglutarate and alanine. Through these enzymatic modifications, glutamate activates several biochemical pathways that stimulate tumor development, including protein and nucleic acid syntheses, epigenetic changes, metabolite exchange between the mitochondria and the cytosol, and the stimulation of antioxidant defense mechanisms [194,195] (Figure 4).

Several studies have indicated that glutamate concentrations have a major impact on the fate of the tumor. For example, in mouse models of lung cancer (LC), the deletion of autophagy-related gene 7 (*Atg7*) decreases macroautophagy, suppresses tumor growth, and promotes cell death [196,197]. LC cells require autophagy to compensate for metabolic stress that is induced by the hypoxic tumor microenvironment, and autophagy promotes the degradation of intracellular components that are necessary for the syntheses of fatty acids, nucleotides, amino acids, and sugars [198]. However, although Atg7 deficiency decreases TCA cycle intermediates (such as glutamate, aspartate, and α-ketoglutarate), supplementations of glutamine or glutamate in the diets of these mice cause a restorative adaptation that increases the survival of LC cells [199]. In addition, NADPH oxidase 4, an enzyme that is highly expressed in LC tumors, promotes glutaminolysis, increases glutamate and glutathione concentrations, and contributes to the survival of LC cells [200]. Disturbances in energy metabolism in cancer cells are reflected in changes in plasma. We recently demonstrated that, in patients with LC or with head and neck cancer (HNC), plasma concentrations of glutamate are strongly increased, compared to those of the healthy population [201]. This study also reported that the measurement of plasma glutamate concentrations has a high diagnostic accuracy in differentiating between patients with LC or HNC and healthy individuals, so it could be a biomarker of these cancers. Another study reported that the plasma glutamate concentration has a high sensitivity and specificity in differentiating between LC and benign lung lesions [202]. Likewise, a high plasma glutamate concentration is associated with low survival [203], and with the presence of neurological complications in patients with LC [204]. Furthermore, glutamate and other glutaminolysis-related products have been proposed as biomarkers of chemotherapy efficacy in patients with oral squamous cell carcinoma [205]. The measurement of plasma concentrations of glutamate via metabolomics, either alone or in combination with other parameters, has demonstrated its usefulness as a biomarker in patients with pancreatic cancer as well [206].

Another alteration that is consistently observed in patients with various types of cancer is a decrease in the plasma concentrations of serine and branched-chain amino acids such as valine, leucine, and isoleucine [201,207,208]. This is due, probably, to an enhanced cellular demand for these amino acids related to increased glutaminolysis. These amino acids are the major nitrogen source for the biosynthesis of glutamine and glutamate [209]. The increased demand for glutamine by actively replicating tumor cells would explain the observed reduced serum concentrations of serine and branched-chain amino acids [210]. LC and HNC patients who had local tumor recurrences or who had died from the disease had higher plasma concentrations of branched-chain amino acids, serine, and other metabolites associated with glutaminolysis [201]. These findings could indicate that a poorer prognosis is associated with a deregulation of glutaminolysis. Several other metabolites have been observed to be related to the clinical characteristics of cancer patients, or to the molecular characteristics of their tumors. Preliminary results have indicated that LC patients with metastases have higher plasma concentrations of β-hydroxybutyrate, whereas those patients with local tumor recurrences had higher values of leucine, valine, and fumarate [201]. Very little is known about the relationships between these metabolites and prognosis of the cancer; β-hydroxybutyrate has been demonstrated to increase the expression of forkhead box O, and the mammalian target of rapamycin. Hence, this factor can stimulate cell growth, proliferation, and longevity [211]. This metabolite also induces the synthesis of metallothioneins, superoxide dismutase, and catalase, thus increasing the antioxidant capacities of cells [212]. Of note is that radiation therapy (RT) has been demonstrated to influence and, to some extent, correct these metabolic disturbances in patients as well as in experimental animals. In mice, RT decreased the hepatic concentrations of choline, O-phosphocholine, and trimethylamine N-oxide, while increasing the concentrations of glutamine, glutathione, malate, creatinine, phosphate, betaine, and 4-hydroxyphenylacetate [213]. A study in patients with glioblastoma demonstrated that post-RT concentrations of 28 metabolites were significantly altered from their pre-RT levels. However, the lack of a control group did not permit identification of the degree of pre-RT metabolic alterations in these patients, as well as whether the effects of treatment normalized or aggravated the alterations [214]. In patients with cervical cancer and radiation-induced acute intestinal symptoms, RT increased the fecal concentrations of α-ketobutyrate, valine, uracil, tyrosine, trimethylamine *N*-oxide, phenylalanine, lysine, isoleucine, glutamine, creatinine, creatine, bile acids, aminohippurate, and alanine, as well as being accompanied by reduced concentrations of α-glucose, *n*-butyrate, methylamine, and ethanol. The authors concluded that metabolomics may be a novel clinical tool for the diagnosis, or therapeutic monitoring, of radiation-induced acute intestinal symptoms [215].

In earlier studies we have reported that the plasma concentrations of the products of glycolysis, TCA cycle, and amino acid metabolism were considerably altered in women with BC, and that RT was associated with a partial rectification of these disturbances [207]. The metabolites that exhibited the strongest pre-RT decreases were serine, valine, leucine, isoleucine, succinate, α-ketoglutarate, glutamate, and malonyl coenzyme A. The parameters that exhibited the strongest increases were pyruvate, aspartate, and aconitate. The majority of these alterations were reversed following RT; the concentrations of lactate, alanine, valine, leucine, isoleucine, proline, malonyl coenzyme A, glycine, succinate, serine, and ketoglutarate were normalized post-RT. The same study also investigated the relationships between metabolic alterations and other concomitant treatments, as well as the clinical characteristics of BC patients. The study reported that adjuvant hormone therapy was associated with lower serum glycine concentrations, and that adjuvant chemotherapy was associated with lower lactate and glutamine concentrations, as well as higher oxaloacetate concentrations, post-RT. In addition, the post-RT plasma concentrations of leucine and isoleucine were significantly lower in estrogen receptor-positive patients than in estrogen receptor-negative patients, and these concentrations were higher in triple-negative patients, in comparison to luminal and Her2 subgroups. Another study in BC patients who received RT after neoadjuvant chemotherapy [208] reported that patients exhibited increased pre-RT concentrations of pyruvate, aspartate, aconitate, and citrate, in conjunction with decreased concentrations of lactate, alanine, valine, leucine, isoleucine, proline, malonyl coenzyme A, glycine, succinate, serine, ketoglutarate, and glutamate. RT largely corrected these alterations, and the improvement was significantly superior in patients who achieved a pathologically complete response than in those patients with partial responses, with serine, proline, and leucine being the parameters with the highest capacity to discriminate between the two groups. These effects of RT on energy metabolism in BC patients cannot be fully extrapolated to other types of cancers. For example, in H&N cancer patients, RT did not ameliorate metabolic alterations but, conversely, was associated with an increase in plasma glutamate and TCA cycle intermediates, such as malate, pyruvate, and succinate [201].

## 8. Infectious Diseases

Bacterial or viral infections cause oxidative stress, inflammation, and metabolic alterations linked to mitochondrial dysfunction as the body’s defense systems respond directly against external agents, or against the interference of these agents in cell homeostasis. Infectious diseases trigger a cascade of reactions in the host, known as the acute-phase response. This response is associated with, amongst others, structural and functional changes in HDL particles that lose their antioxidant and anti-inflammatory properties [216,217,218]. Many studies have identified decreased serum PON1 activities and increased CCL2 concentrations in infectious diseases. A proteomic study reported decreased PON1 expression in patients with sepsis, compared to healthy individuals [219]. We recently observed that hospitalized patients carrying an indwelling central venous catheter [220] or a urinary catheter, with catheter-associated asymptomatic bacteriuria [221] had decreased serum PON1 activities and increased CCL2 concentrations. Based on our findings, we proposed the measurement of the circulating levels of these molecules as useful markers of acute concomitant infection. We found similar alterations in patients with severe sepsis admitted to the Intensive Care Unit; the alterations tended to normalize when the sepsis was corrected [222,223]. Serum PON1 activity was found to be low in several studies in patients infected with *Helicobacter pylori*; this alteration may play a role in the high predisposition of these patients to develop atherosclerosis and CVD [224,225,226,227,228]. Decreased serum PON1 activities were also observed in patients infected by *Brucella* [229], or *Mycobacterium tuberculosis* [230], together with increased release of pro-inflammatory cytokines.

Viral infections are associated with similar disruptions. Patients with human immunodeficiency virus (HIV) infection have decreased circulating levels of HDL-cholesterol and PON1 activity, and increased CCL2 [231,232,233,234,235,236]. These patients often develop pro-atherogenic metabolic alterations, which can be explained by the infection itself, or by the effects of antiretroviral therapies [237,238,239]. Higher PON1 activities and lower CCL2 concentrations have been related to higher CD4+ T lymphocyte counts, which indicate a better immunological status [233]. Recent studies observed that CCL2 participates in the onset and development of neurocognitive disorders in HIV-infected patients [240,241,242,243]. Similar alterations in PON1 and CCL2 levels have been reported in patients with hepatitis B [244,245,246], hepatitis C [247,248], and dengue [249]. Although data on alterations in energy metabolism in HIV infection are scarce, some studies using metabolomic methods showed significant associations between CCL2, sphingomyelins, phospholipids, and triglycerides [250], Additionally, the alterations in glutaminolysis and pro-inflammatory molecules appear to be the metabolomic signature of late immune recovery post-treatment [251]. Moreover, increased plasma glutamine levels have been found to be related to CVD in these patients [252].

In this article, which has “A 2021 update” in the title, we must not miss the opportunity of commenting on the special circumstances in which we currently find ourselves, i.e., the COVID-19 pandemic. Available data on this issue are very preliminary. A proteomic study found decreased PON1 expression in the HDL of COVID-19 patients [253]; the role of PON1 in COVID-19 may be different depending on whether the enzyme is present in the circulation or within the cells. Purified native HDL with intact PON1 elicits a potent antiviral effect against SARS-CoV-2 in cultured monocyte cells, while glycated HDL, with inactive PON1, lost its antiviral activity [254]. However, an in silico study reported that PON1 enhances the action of ACE2, the main cell receptor of SARS-CoV-2 [255], and the inhibition of PON1 activity has been described as being a potent inhibitor of vaccinia virus early protein synthesis and viral mRNA methylation in mice [256], suggesting that intracellular PON1 is important in limiting the translation of viral proteins and virus replication. High CCL2 concentrations have been observed in the circulation [257,258,259,260,261,262] in bronchoalveolar lavage fluid [263], and in lung tissue of COVID-19 patients [264]. One study reported that CCL2 expression increases rapidly in the early acute phase of infection and then progressively decreases as the disease advances [265]. The effects of this infection on mitochondrial function and energy metabolism deserve further research, because preliminary data suggest they could be very clinically relevant [266,267].

## 9. Final Remarks

We make no pretense at an exhaustive review of all the articles that demonstrate a participation of PON1, CCL2, and metabolic alterations in all known diseases. We have not addressed neurological, autoimmune, or kidney diseases, or conditions caused by food poisoning or xenobiotics. Nor have we mentioned the alterations produced in these parameters by surgical procedures, nor the successful (or unsuccessful) attempts to modulate these pathways through dietary or pharmacological interventions. Indeed, such a task is beyond the remit of a review article. Rather, we sought to highlight some of the common pathophysiological mechanisms of many communicable and/or non-communicable diseases, particularly with respect to the important roles that PON1 and CCL2 play in metabolic changes linked to mitochondrial dysfunction. Our goal has been to encourage interested readers to undertake their own research in a field that we believe will have transformational scientific and clinical implications in the near future.
